# Artemisinin and its derivatives can significantly inhibit lung tumorigenesis and tumor metastasis through Wnt/β-catenin signaling

**DOI:** 10.18632/oncotarget.8920

**Published:** 2016-04-22

**Authors:** Yunli Tong, Yuting Liu, Hongming Zheng, Liang Zheng, Wenqin Liu, Jinjun Wu, Rilan Ou, Guiyu Zhang, Fangyuan Li, Ming Hu, Zhongqiu Liu, Linlin Lu

**Affiliations:** ^1^ School of Pharmaceutical Sciences, Southern Medical University, Guangzhou, Guangdong, 510515, China; ^2^ International Institute for Translational Chinese Medicine, Guangzhou University of Chinese Medicine, Guangzhou, Guangdong, 510006, China; ^3^ Department of Pharmacological and Pharmaceutical Sciences, College of Pharmacy, University of Houston, Houston, Texas, 77030, USA

**Keywords:** non-small-cell lung cancer, artemisinin, derivative, Wnt/β-catenin

## Abstract

Non-small-cell lung cancer (NSCLC) is the most prevalent malignancy worldwide given its high incidence, considerable mortality, and poor prognosis. The anti-malaria compounds artemisinin (ART), dihydroartemisinin (DHA), and artesunate (ARTS) reportedly have anti-cancer potential, although the underlying mechanisms remain unclear. In this work, we used flow cytometry to show that ART, DHA, and ARTS could inhibit the proliferation of A549 and H1299 cells by arresting cell cycle in G1 phase. Meanwhile, tumor malignancy including migration, invasion, cancer stem cells, and epithelial–mesenchymal transition were also significantly suppressed by these compounds. Furthermore, ART, DHA, and ARTS remarkably decreased tumor growth *in vivo*. By using IWP-2, the inhibitor of Wnt/β-catenin pathway, and Wnt5a siRNA, we found that ART, DHA, and ARTS could render tumor inhibition partially dependent on Wnt/β-catenin inactivation. These compounds could strikingly decrease the protein level of Wnt5-a/b and simultaneously increase those of NKD2 and Axin2, ultimately resulting in β-catenin downregulation. In summary, our findings revealed that ART, DHA, and ARTS could suppress lung-tumor progression by inhibiting Wnt/β-catenin pathway, thereby suggesting a novel target for ART, DHA, and ARTS in cancer treatment.

## INTRODUCTION

Lung cancer remains the leading cause of cancer-related deaths worldwide because of its high incidence and mortality [1, 2]. Non-small-cell lung cancer (NSCLC), which accounts for approximately 85% of all lung cancer cases, is composed of various histological subtypes, among which adenocarcinomas are responsible for almost 40% diagnoses [3, 4]. Given that 67.6% of lung-cancer patients have been diagnosed in advanced stages, chemotherapies including molecular-drug-targeted mutations of epidermal growth factor receptor (EGFR) remain the best and standard strategies for lung cancer treatments after surgery. However, drug resistance, subsequent metastasis, and accumulated toxicity to normal cell and immune system limit the clinical benefit of chemotherapies and the median survival to 1 year [4, 5]. Therefore, utilizing food-derived, plant-derived, or pharmaceutical interventions with broad effectiveness and tolerable side effects to preventtumor malignancy and invasion is a promising approach for metastasis reduction, and overall survival improvement [6].

Wnt/β-catenin signaling pathway, prominently activated in NSCLC, plays a critical role in lung tumorgenesis and metastasis and also mediates drug resistance [7]. Wnt5-a/b, defined as “destruction complex” along with Frizzled receptors, lipoprotein-receptor-related protein (LRP), dishevelled (Dvl), and scaffold protein axis inhibition protein (Axin), is a prerequisite for Wnt/β-catenin activation. Conversely, Axin2 [8] and *Drosophila* naked cuticle (NKD2) [9], which are negative regulators in canonical Wnt signaling, could suppress β-catenin by inhibiting its transcription while promoting ubiquitination [10]. With increased β-catenin translocated from cytoplasma to nucleus [11, 12], the transcriptional activities of various downstream genes including cyclin D1, oct3/4, sox2, and nanog are strikingly stimulated [13–15]. The abundant expressions of sox2, oct3/4, and nanog represent the pluripotency of cancer stem cells (CSCs) [14, 16], and cyclin D1 serves as a primary mediator in cell cycle maintenance [17]. Hence, the activation of Wnt/β-catenin may result in increased malignancy and continuous tumor cell proliferation. Moreover, by decreasing the protein expression of E-cadherin, while increasing that of N-cadherin and vimentin, epithelial–mesenchymal transition (EMT) could also be promoted by β-catenin in lung cancer metastasis [18]. The schematic of Wnt/β-catenin pathway is shown in Figure 7.

Some epidemiological studies have revealed that various dietary compounds such as green tea catechins, lycopene, selenium, and vitamin A could be regard as chemopreventive agents for lung cancer recurrence and metastasis [6]. Especially, some phytochemical agents including resveratrol [19], curcumin [20], and wogonin [21] also reportedly have anti-tumor efficacy by suppressing Wnt/β-catenin pathway. However, partially due to low pulmonary bioavailability and unclear targets of these alternative therapeutic agents, clinical trials for NSCLC chemoprevention have yielded negative results [22, 23]. Thus, identifying a component with better physicochemical property, less toxicity, and certain molecular targets can aid research on lung cancer treatment and survival improvement.

*Artemisia annua* L., a traditional medicine used for more than 2000 years, has significant efficacy against malaria, parasitic diseases, and fibrosis with low toxicity [24]. Artemisinin (ART), along with its derivatives such as artesunate (ARTS) and dihydroartemisinin (DHA), are the safe, predominant compounds in anti-malarial treatment [25–28]. Meanwhile, the conventional administration of ART, DHA, and ARTS depends on oral delivery, suggesting the high relative bioavailability of these agents [29]. According to some pharmacokinetic studies, the strong amphipathic characteristics of ARTS and DHA lead to the clearance time of 6 h after a single intravenous injection [30]. Among three compounds, DHA had the highest relative bioavailability (>80%) after oral intake in rats and humans [31, 32].

Recent studies have revealed that these three components markedly inhibit tumor growth and metastasis of NSCLC with preferential cytotoxicity in tumor cells even in micromoles [33–36]. Other studies have demonstrated that DHA induced lung cancer cell apoptosis by inhibiting the phosphorylation of p70 S6 kinase 1 in an mTORC1-dependent manner [37]. Also reports have shown that ART notably suppressed tumor invasion and metastasis in NSCLC cells. Whereas whether ART, DHA, and ARTS could suppress tumors by inhibiting Wnt/β-catenin pathway, as well as the precise molecular targets involved, remains unclear. Additionally, the potential of these herbal medicines with low toxicity and high anti-tumor effectiveness to become chemopreventive candidates is under investigation.

In the current study, by utilizing an A549 xenograft model, the anti-tumor efficacy of ART, DHA, and ARTS were addressed *in vivo*. In addition, the anti-proliferation and anti-metastasis effects of ART and its two derivatives were further evaluated in two different lung cancer cell lines, including A549 (carcinoma *in situ*) and H1299 (adenocarcinoma from metastasis nodule). Furthermore, in Wnt/β-catenin pathway, we also investigated the underlying mechanism and explored drug-sensitive targets. Considering the low cytotoxicity and high bioavailability of ART, DHA, and ARTS, determining the effect of these compounds on Wnt/β-catenin pathway may provide information on their potential as perfect chemopreventive candidates.

## RESULTS

### ART, DHA, and ARTS suppressed cell viability and induced G1 arrest by inhibiting cyclin D1 in A549 and H1299

**Figure 1:**
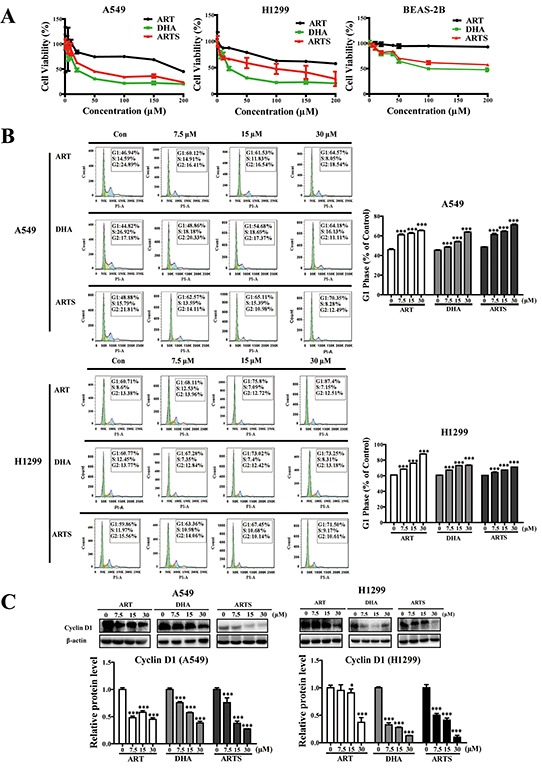
Effects of artemisinin (ART), dihydroartemisinin (DHA), and artesunate (ARTS) on cell viabilities and cell cycle progression **A.** Relative cell viabilities of A549, H1299, and BEAS-2B treated with ART, DHA, and ARTS (0-200 μM). **B.** Percentages of A549 and H1299 cells in the G1 phase after treatment with or without ART, DHA and ARTS. **C.** Effects of ART, DHA, and ARTS on expression of cyclin D1. Each column represented the mean ± standard deviation (SD). *p < 0.05, ***p < 0.001, compared with the control group (Con, control).

MTT and flow cytometry assays were conducted to investigate the effects of ART, DHA, and ARTS on the cell fate of A549 and H1299 cells. After treating cells with ART, DHA, and ARTS (0–200 μM) for 48 h, cell viabilities were significantly suppressed. Additionally, the sensitivities of tumor cell inhibition among these compounds decreased in the order DHA > ARTS > ART. However, ART, DHA, and ARTS treatments did not effectively inhibit the growth of BEAS-2B (normal human bronchial epithelial cells) up to 70 μM because IC_50_ was observed only in DHA at 76.95 μM (Figure 1A). Furthermore, with increased concentrations of ART, DHA, and ARTS, tumor cells arrested in G1 phase significantly accumulated. Especially, ART at 30 μM markedly increased the percentage of tumor cells in G1 phase with 17.63% ± 0.67% (*p* < 0.001) and 26.69% ± 0.68% (*p* < 0.01) in A549 and H1299, respectively (Figure 1B). However, none of ART, DHA, and ARTS could notably alter the cell cycle of BEAS-2B (Supplementary Figure S1). Cyclin D1, the vital regulator of G1 progression, of which protein level was significantly reduced by ART, DHA, and ARTS both in A549 and H1299 cells. Yet, cyclin D1 decreased in a dose-dependent manner only in DHA and ARTS treatments. Additionally, ARTS also exerted greater suppressive effect on cyclin D1 expression than ART and DHA (Figure 1C).

### Cell invasion and migration were inhibited by ART, DHA, and ARTS

**Figure 2:**
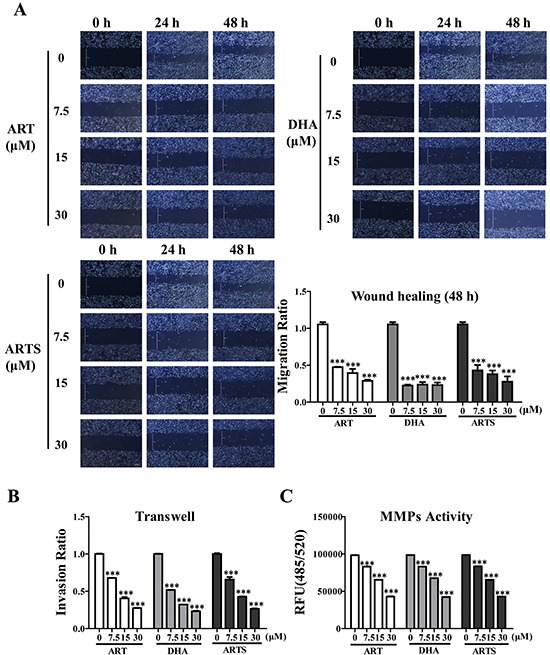
Effects of ART, DHA, and ARTS on cell migration and invasion **A.** Representative photomicrographs of initial and final wounds at 24 and 48 h were shown at 100× magnification. Cell migration distance data were presented as the means ± SD of three independent experiments. **B.** Cell invasion data in A549 cell line shown were the means ± SD. **C.** Inhibitory effects of ART, DHA, and ARTS on MMPs activities were shown. Each bar was representative of the mean ± SD. (***p < 0.001, compared with the control group).

Wound healing assay was used to evaluate migration in A549 and H1299 cells. ART, DHA, and ARTS significantly inhibited lung tumor cell migration in a dose-dependent manner. Among all three compounds, DHA at 30 μM showed the most effective migration suppression with 27.1% ± 3.52% compared with the control group (Figure 2A; *p* < 0.001). Additionally, transwell coated with matrigel was used to explore the invasive ability of lung tumor cells. Compared with abundant cells translocated to the underside of well in the control treatment, cell invasion activities were dose-dependently decreased by ART, DHA, and ARTS. Especially in ART treatments, cell invasion significantly decreased by 32.19% ± 0.15% (*p* < 0.001), 58.77% ± 1.79% (*p* < 0.001), and 67.25% ± 0.22% (*p* < 0.001), respectively, with increased concentrations (7.5, 15, and 30 μM; Figure 2B). Meanwhile, matrix metalloproteinase (MMPs) activities were analyzed by MMPs activity assay kit to further determine the invasion capability of ART, DHA, and ARTS. All ART, DHA, and ARTS treatments could markedly inhibit MMPs activities in a dose-dependent manner (Figure 2C).

### ART, DHA, and ARTS suppressed EMT and CSCs

**Figure 3:**
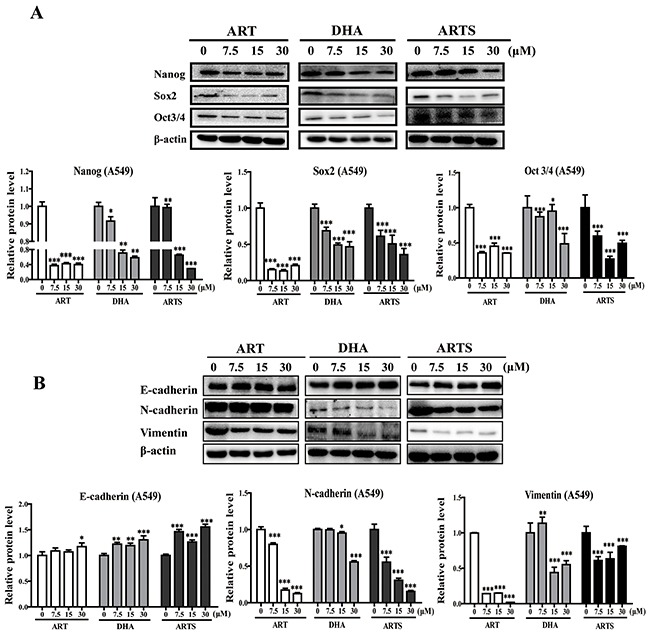
Epithelial–mesenchymal transition (EMT) and cancer stem cells (CSCs) were regulated by ART, DHA, and ARTS in A549 in vitro **A.** Expression of CSCs regulatory proteins was determined by Western blot in A549 with antibodies against nanog, sox2, and oct3/4. **B.** Western blot analysis was performed for E-cadherin, N-cadherin, and vimentin. Data shown were the means ± SD. *p < 0.05, **p < 0.01, ***p < 0.001, compared with the control group (Con, control).

CSCs and EMT are two key factors affecting tumor metastasis, including migration and invasion. Whether the anti-metastasis effects of ART, DHA, and ARTS are associated with these two aspects were analyzed by Western blotting. Results showed that the expression levels of CSC markers including nanog, sox2, and oct3/4, as well as EMT-related proteins such as N-cadherin and vimentin, were all significantly inhibited by ART, DHA, and ARTS treatments, whereas those of E-cadherin either in A549 (Figure 3) or H1299 cells (Supplementary Figure S2) increased. Among the three compounds, ART exerted the greatest inhibitory effects, especially in the expression of nanog and sox2 in A549 cells, and the percentages of decrease at 30 μM were 65% ± 0.26% (*p* < 0.001) and 80% ± 1.92% (*p* < 0.001), respectively (Figure 3A). Similarly, the protein levels of N-cadherin and vimentin were remarkably down-regulated by ART (Figure 3B). However, in H1299 cells, most increased E-cadherin expression and decreased CSCs markers were observed in ARTS treatments (Supplementary Figure S2B), indicating that the effective response of ART, DHA, and ARTS may slightly vary in tumor cells with different histological features.

### ART, DHA, and ARTS depressed Wnt/β-catenin signaling pathway

**Figure 4:**
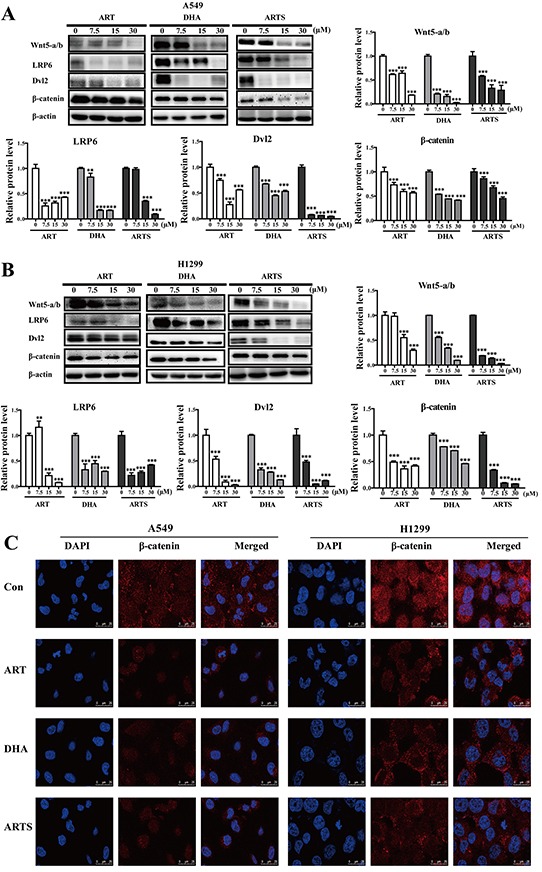
ART, DHA, and ARTS decreased Wnt5-a/b-triggered suppression of downstream proteins *in vitro* **A.** A549 cells were harvested, and Western blot analysis was conducted for Wnt5-a/b, LRP6, Dvl2, and β-catenin. **B.** H1299 cells were harvested, and Western blot analysis was conducted for Wnt5-a/b, LRP6, Dvl2, and β-catenin. Data shown were the means ± SD. **p < 0.01, ***p < 0.001, compared with the control group. **C.** Representative confocal imaging of nuclei (blue), β-catenin (red), and nuclei/β-catenin double-stained cells at the level of β-catenin cytoplasm and nuclear localization after 48 h of ART, DHA, and ARTS treatments. Nuclei were counterstained with DAPI (scale bar: 25 μm).

To determine whether Wnt/β-catenin pathway was involved in the anti-tumor and anti-metastasis effects of ART and its two derivatives, key proteins in Wnt/β-catenin pathway were analyzed by Western blotting. In both A549 and H1299 cells, most dramatic reduction were found on the expressions of Wnt5-a/b after ART, DHA, and ARTS treatments, even at 7.5 μM (Figure 4A and 4B). Furthermore, the subsequent proteins in Wnt/β-catenin signaling pathway were also altered remarkably by ART and its two derivatives. For example, LRP6 significantly decreased by 16.80% ± 1.14% after DHA (30 μM) treatment in A549 cells (Figure 4A; *p* < 0.001), whereas the protein levels of Dvl2 were markedly reduced by ARTS at 15 μM by 6.50% ± 0.64% (*p* < 0.001) and 3.58% ± 0.42% (*p* < 0.001), respectively, in A549 and H1299 cells (Figure 4A and 4B). Moreover, in those cell lines, ART, DHA, and ARTS treatments significantly decreased the protein of β-catenin accumulated either in cytoplasm or nucleus (Figure 4C).

### Suppression of ART, DHA, and ARTS in proliferation and metastasis could partially depend on Wnt/β-catenin pathway inactivation

**Figure 5:**
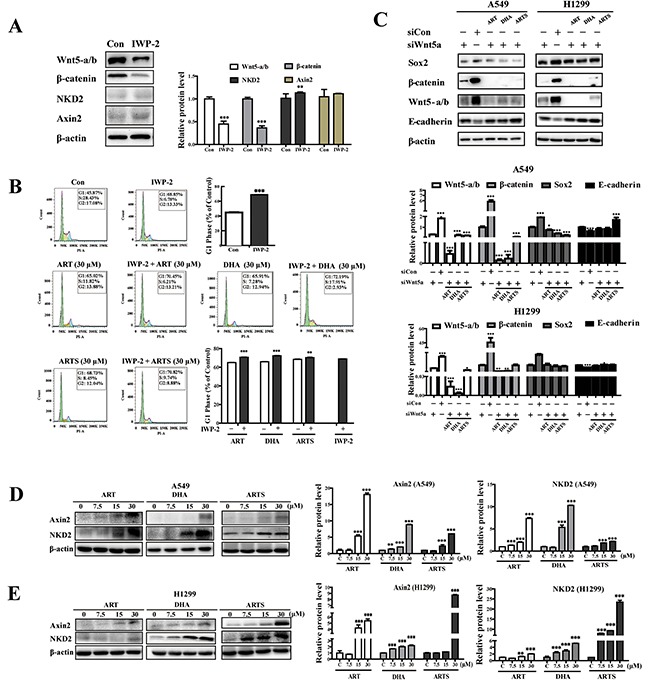
ART, DHA, and ARTS were partially dependent on Wnt/β-catenin signaling **A.** A549 cells were harvested, and Western blot analysis was conducted for β-catenin, Wnt5-a/b, NKD2, and Axin2 with or without IWP-2. **B.** Percentage of A549 cells in the G1 phase with 0 and 30 μM ART, DHA, and ARTS treatments accompanied with or without IWP-2. **C.** Cells were transfected with Wnt-5a siRNA or control siRNA before ART, DHA, and ARTS (30 μM) treatments. Wnt5a, β-catenin, sox2, and E-cadherin protein levels were detected by Western blot analysis. **D.** A549 cells were harvested, and Western blot analysis was performed for Axin2 and NKD2. **E.** H1299 cells were harvested, and Western blot analysis was conducted for Axin2 and NKD2. The data represent mean ± SD. *p < 0.05, **p < 0.01, ***p < 0.001, compared with the control group (Con, control).

To determine whether the anti-tumor effects of ART and its derivatives depended on Wnt/β-catenin pathway, IWP-2, Wnt/β-catenin pathway inhibitor and Wnt5a siRNA were used considering the important role of Wnt5-a/b and of which most protein reduction were observed previously. The expressions of Wnt5-a/b and β-catenin in A549 cells were dramatically suppressed by IWP-2, whereas those of NKD2 and Axin2 were not altered (Figure 5A). However, pretreated with IWP-2, the G1 arrest induced by ART, DHA, and ARTS were enhanced in A549 cells. While interestingly, compared with IWP-2 treatment, no significant differences were observed in treatments of ART and its derivatives combined with IWP-2 (Figure 5B). Moreover, as expected, Wnt5-a/b knockdown consequently suppressed the protein expressions of sox2 and β-catenin but increased that of E-cadherin. However, with Wnt5a knockdown, ART, DHA, and ARTS still exerted anti-metastasis effect by decreasing sox2 while increasing E-cadherin (Figure 5C). Therefore, with or without Wnt5a, the irreplaceable and sustained suppression of Wnt/β-catenin pathway occurred, suggesting that the anti-tumor effects of ART and its derivatives only partially depended on Wnt5-a/b inactivation. On the other hand, Axin2 and NDK2, two negative regulators in canonical Wnt/β-catenin signaling pathway, were simultaneously explored. In A549 cells, ART at 7.5, 15, and 30 μM significantly increased the protein level of NKD2 by 132.38% ± 4.60% (*p* < 0.001), 198.83% ± 12.25% (*p* < 0.001), and 734.09% ± 20.14% (*p* < 0.001), respectively (Figure 5D). Similar results were observed in H1299 cells (Figure 5E). Additionally, the expressions of Axin2 were also notably increased by ART in A549 cells and by ARTS in H1299 cells.

### ART, DHA, and ARTS restrained lung cancer growth in A549 xenograft mice

**Figure 6:**
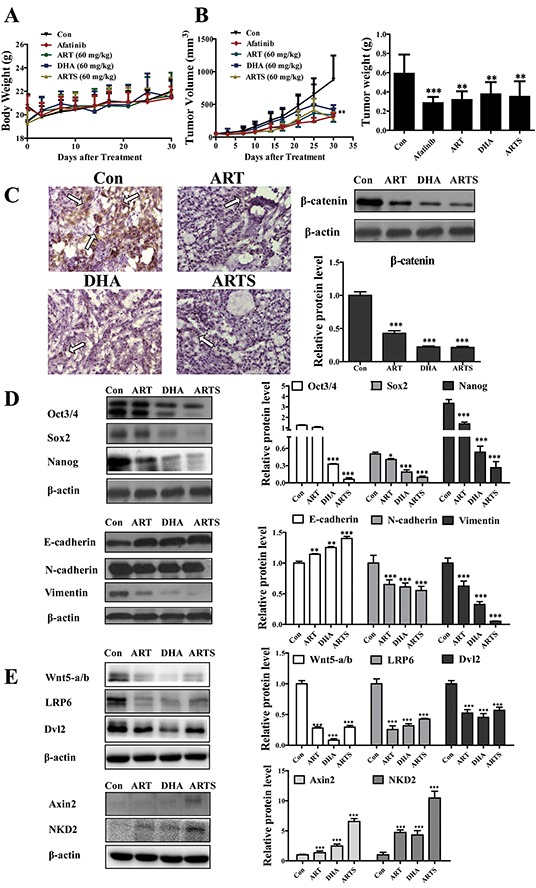
ART, DHA, and ARTS suppressed cell proliferation in A549 xenograft mice model by inhibiting Wnt/β-catenin, EMT and CSCs *in vivo* **A.** Body weights of mice during the ART, DHA, and ARTS treatments. **B.** Tumor volume was measured during the experiment, and tumor weights of A549 xenografts were measured after four weeks of treatment. **C.** High magnification (400×) of tumor sections treated with or without ART, DHA, and ARTS. A high signal corresponding to β-catenin protein in control group was detected (bar: 50 μm). Protein expression of β-catenin in lysates of tumor tissue from mouse was detected by Western blot. **D.** Protein expressions of markers of EMT and CSCs, namely, E-cadherin, N-cadherin, vimentin, nanog, sox2, and oct3/4 were detected by Western blot. **E.** Protein expressions of Wnt5-a/b, LRP6, Dvl2, Axin2, and NKD2 in lysates of tumor tissues from mice were detected by Western blot. Data shown were the means ± SD, *p < 0.05, **p < 0.01, ***p < 0.001, compared with the control group (Con, control).

The A549 xenograft model was employed to further explore the effects and toxicity of ART, DHA, and ARTS *in vivo*. ART, DHA, and ARTS, as well as afatinib, did not cause significant body weight loss in mice (Figure 6A). While the tumor volumes also remarkably shrank after ART, DHA, and ARTS gavage, and maximum inhibition (41.04%) was observed after ARTS treatment (Figure 6B; *p* < 0.01). In addition, tumor weights were significantly reduced as well in the ART, DHA, and ARTS groups compared with that in the control group (Figure 6B; *p* < 0.05).

To verify the observation *in vitro*, EMT and CSC markers and Wnt associated proteins were examined in tumor tissues from xenograft mice. Immunostaining staining was performed to confirm β-catenin expression *in situ*. Compared with the control group, β-catenin protein level was significantly decreased by approximately 50% in tumor tissues after ART, DHA, and ARTS treatments (Figure 6C). Furthermore, the expression levels of oct3/4, sox2, and nanog were respectively decreased by 5.10% ± 0.25% (*p* < 0.001), 19.30% ± 0.19% (*p* < 0.001), and 7.92% ± 0.15% (*p* < 0.001) in ARTS treatment. By contrast, the inhibition effect of ART was less significant than those of ARTS and DHA. Similarly, ARTS and DHA remarkably decreased the expression of vimentin more than that observed in ART treatment (Figure 6D). Additionally, in tumor tissues collected from A549 xenograft model, ART and its derivatives effectively reduced Wnt5-a/b, LRP6, and Dvl2 but significantly increased both NKD2 and Axin2 (Figure 6E; *p* < 0.001).

## DISCUSSION

For lung cancer patients in stages II and IIIa, surgical resection complemented with target therapeutics may achieve a good prognosis [38]. However, chemotherapies, act as a double-edged sword, could also simultaneously lead to numbers of side effects, such as diarrhea, headache, and ototoxicity when exert anti-tumor effectiveness [39, 40]. Research has demonstrated that ART has neurotoxicity and embryotoxicity in various species, although much more clinical studies and meta-analyses have found no serious side effects of ART, DHA, and ARTS [41, 42]. While the toxicity of ART in mammal pneumonocytes also remains unconfirmed [43, 44]. According to our MTT results, among the three compounds, ART had the lowest cytotoxicity to lung tumor cells, whereas its metabolite DHA exerted highest (Figure 1A). More strikingly, compared with the cell viability suppression and cell cycle arrest observed in tumor cells, ART, DHA, and ARTS had minor toxicity on the growth of normal human bronchial epithelial cells, without even slight influencing the cell cycle of BEAS-2B (Supplementary Figure S1). These results suggested that ART and its derivatives were potential excellent chemopreventive agents with almost no cytotoxicity to normal pneumonocytes.

Clinical studies have shown that although target therapeutic agents could prolong progression-free survival to 9.7 months, response rate varied due to individual differences [45]. For example, erlotinib, a sensitive EGFR tyrosine kinase inhibitor, could arouse only a 58% response in NSCLC patients [46]. By examining cell viabilities in lung carcinoma cells (A549) and non-small lung cancer cells (H1299), respectively, we found that regardless of histological clarification, ART, DHA, and ARTS may exert broad tumor suppressions *in vitro*. In both these two lung cancer cell lines, ART, DHA, and ARTS suppressed the proliferation of tumor cells by arresting their cycle in G1 phase and simultaneously decreasing cyclin D1 expression (Figures 1A–1C). The non-dose-dependent down-regulation of cyclin D1 may be ascribed to other regulators participated in G1 phase, such as cyclin E, cdk2, and cdk4 [47, 48].

Tumor-metastasis inhibition is a main criterion in determining whether a compound could be an effective chemopreventive agent [49]. Our data showed that ART, DHA, and ARTS significantly suppressed both cell migration and invasion *in vitro* either in A549 or H1299 cells (Figures 2A–2C). Furthermore, according to mechanism studies, the successful initiation of metastatic progression known as “metastatic colonization” is accomplished only by a minority of cancer cells, such as CSCs [16]. Thus, we evaluated the protein expression of the CSC markers nanog, sox2, and oct3/4 in the present work. As shown in Figure 3A and Supplementary Figure S2A, ART, DHA, and ARTS decreased the expression of these CSCs markers in lung cancer cells, even in tumor tissues *in vivo* (Figure 6D), suggesting an inhibition of these compounds on CSCs pluripotency. Simultaneously, the EMT markers N-cadherin and vimentin were markedly inhibited by ART, DHA, and ARTS treatments, whereas E-cadherin was significantly increased either *in vitro* (Figure 3B and Supplementary Figure S2B) or *in vivo* (Figure 6D). Therefore, with notable anti-metastasis effectiveness of ART and its derivatives strengthens their potentials as chemopreventive candidates.

To further explore the anti-tumor effectiveness of ART, DHA, and ARTS *in vivo* and also the implied risk of toxicity after biotransformation, A549 xenograft model was applied. Compared with the control group, without altering the body weight of mice, ART, DHA, and ARTS significantly decreased tumor volume and tumor weight (Figure 6A and 6B). Afatinib, an outstanding chemotherapeutic agent, not served as a positive control drug in our experiment, but only be chosen because of its oral administration regardless of its EGFR inhibitory effects. Subsequently, by staining tumor tissues, the protein expression of β-catenin decreased after ART, DHA, and ARTS treatments, indicating that β-catenin inactivation may be involved in their anti-tumor effects.

**Figure 7:**
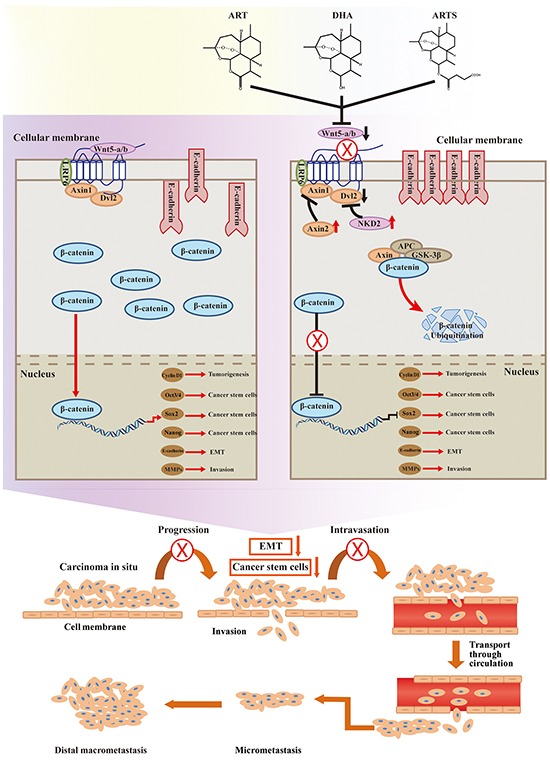
Inhibition of canonical Wnt/β-catenin signaling Canonical Wnt signaling was inhibited by ART, DHA, and ARTS. Besides inhibiting Wnt signaling through their interaction with Frizzled, secreted frizzled-related proteins may also inhibit the EMT. β-catenin can inhibit the transcription of CSCs and reduce the levels of cyclin D1. Axin2 and NKD2 are two negative regulators of Wnt/β-catenin signaling. Axin2 and NKD2 expression was negative in resected NSCLC. (X, ⊥, suppression of this progress. ↓, decrease of protein expression. ↑, increase of protein expression.)

Wnt/β-catenin pathway plays a critical role in every stage of cancer progression, including initiation, development, and metastasis [50, 51]. Furthermore, because of the potential to maintain cancer stem cells and EMT, it is regarded as an attractive target for cancer prevention [52]. In the present study, we observed that ART, DHA, and ARTS inhibited the expression level of Wnt5-a/b, down-regulated those of LRP6 and Dvl2, and subsequently reduced those of downstream genes mediated by β-catenin (i.e., nanog, sox2, oct3/4, and cyclin D1) in two lung cancer cell lines (Figure 4). Additionally, the inhibition of ART, DHA, and ARTS in Wnt/β-catenin activation was also confirmed in tumor tissues collected from xenograft model *in vivo* (Figure 6E). Given the significant inhibition of Wnt/β-catenin pathway in ART, DHA, and ARTS treatments, we further determined the molecular target of ART, DHA, and ARTS using Wnt5a siRNA as well as IWP-2, specific inhibitor of Wnt/β-catenin pathway [53]. Compared with IWP-2, no significant difference in G1 arrest was observed in IWP-2 combined with ART, DHA, and ARTS, respectively, indicating that Wnt/β-catenin signaling pathway was probably the most vital pathway influenced by ART, DHA, and ARTS (Figure 5B). Moreover, Wnt5a silencing via siRNA did not fully abrogate the effect of ART, DHA, and ARTS on Wnt/β-catenin pathway and downstream proteins, suggesting although very important, Wnt5a was still not the only target for ART and its derivatives (Figure 5C). Other than that, Axin2 and NKD2, negative regulators in the canonical Wnt/β-catenin signaling pathway [9, 54], were significantly increased as well by ART, DHA, and ARTS. Thus, apart from Wnt5a, negative mediators were also potential targets of ART, DHA, and ARTS (Figure 5D and 5E). Schematic representation of targets, mediated by ART, DHA, and ARTS, in Wnt/β-catenin pathway was shown in Figure 7.

Considering the irreversible drug resistance and cytotoxicity of chemotherapy, its benefits in the treatment of and life-quality improvement of early-stage lung-cancer patients remain controversial. By affecting multiple targets in Wnt/β-catenin pathway, a developmental pathway influencing the clinical outcomes of early-diagnosed lung cancer patients, ART, DHA, and ARTS markedly suppressed lung tumorigenesis and exerted a perfect effect on inhibiting tumor metastasis. Therefore, ART and its derivatives are excellent chemopreventive candidates with high bioavailability, low toxicity, and significant anti-tumor efficacy [29]. Further studies should be conducted in chemical-induced lung cancer models such as benzopyrene (BaP)-mediated lung adenocarcinoma in A/J mice to evaluate and confirm the chemopreventive effect of ART and its derivatives.

## MATERIALS AND METHODS

### Chemicals and reagents

ART, DHA, and ARTS were purchased from Dalian Meilun Biotech Co., Ltd (Dalian, China). IWP-2 and 3-(4, 5-dimethylthiazol-2-yl)-2, 5-diphenyltetrazolium bromide (MTT) were obtained from Sigma-Aldrich. Primary antibodies for LRP6, Axin2, Dvl2, NKD2, and cyclin D1 were purchased from Cell Signaling Technology, Inc. And β-actin, oct3/4, nanog, sox2, E-cadherin, N-cadherin, and vimentin primary antibodies, secondary HRP-conjugated antibodies, Wnt5a siRNA and respective control siRNA were purchased from Santa Cruz Biotechnology. Primary antibodies for Wnt5a and β-catenin IHC staining were purchased from Abcam. Western blot detection reagents were obtained from Bio-Rad Laboratories. Amplite™ Universal Fluorimetric MMPs Activity Assay Kit was purchased from AAT Bioquest. Propidium iodide (PI) staining and matrigel were purchased from Biosciences.

### Cell lines

The human lung cancer cells (A549 and H1299) and bronchial epithelial cells (BEAS-2B) were purchased from ATCC (Manassas, Virginia, USA). A549 and H1299 cells were cultured in RPMI-1640 medium, while BEAS-2B cells were maintained in BEGM medium supplemented with 10% FBS and 1% penicillin–streptomycin solution in a humidified atmosphere at 37°C with 5% CO_2_. And BEAS-2B was cultured in BEGM medium.

### Animals and xenograft model

Investigation has been conducted in accordance with the ethical standards and according to the Declaration of Helsinki and according to national and international guidelines and has been approved by the authors’ institutional review board.

Animal experiments, approved by the Guangzhou University of Chinese Medicine Animal Care and Use Committee (Guangzhou, China), have been conducted in accordance with the ethical standards and national guidelines. Female Balb/c-nude mice (4-6 weeks, 18-20g) were purchased from Laboratory Animal Center of Sun Yat-Sen University (Guangzhou, China), and then subjected to subcutaneously injection with A459 cells (2 × 10^6^, suspended in PBS) in each right flank. Tumor volume (TV) was defined based on two dimensions (L, long diameter; W, wide diameter) measured by calipers, and calculated as formula: TV (mm^3^) = (L × W^2^)/2. When the tumors reached a mean volume of 50 mm^3^, all mice were randomized into following groups: gavage control (sterilized coin oil), afatinib (5 mg/kg/d), ART (60 mg/kg/d), DHA (60 mg/kg/d), and ARTS (60 mg/kg/d). Body weights were recorded twice a week. Treatments were administered orally five times a week for four weeks. At the end point, sacrificed the mice, and removed the tumors for following assays.

### Immunohistochemistry

Tumor tissue specimens were fixed in neutral formalin and embedded in paraffin after collection from the sacrificed mice. Then tissue sections were cut and dewaxed, they were incubated with 0.01 M natrium citricum for antigen retrieval. The slides were rinsed in phosphate-buffered saline and incubated overnight at 4°C with diluted anti-β-catenin antibodies. Following steps were performed using the immunostaining kit (BOSTER Biological Technology) according to the manufacturer's instructions.

### MTT assay

The MTT assay was used to evaluate the cell viability. 2 × 10^4^ cells/ml of A549, H1299, and BEAS-2B cells were seeded into 96-well plates and cultured with different doses of ART, DHA, and ARTS for up to 48 h. At the end of treatment, 0.5 mg/ml of MTT was added to the samples and incubated for 4 h. Then the supernatants were discarded and coloured formazan crystals dissolved with 150 μl/well of DMSO. Then OD values were read by using a microplate reader Victor X3 (PerkinElmer, Waltham, MA, USA) at 570 nm.

### Cell cycle analysis

1 × 10^6^ cells/ml of A549, H1299, and BEAS-2B were treated with vehicle or ART, DHA, and ARTS, for up to 48 h. For cell cycle analysis, cells were harvested, fixed with 70% cold ethanol, incubated with RNase, and stained with propidium iodide. Cell cycle was detected by flow cytometry (BD Biosciences, San Diego, CA, USA) and analyzed by FlowJo v7.6 software.

### Wound healing assay

Wound healing assay was conducted to examine the capacity of cell migration. Cells were treated with 0, 7.5, 15, or 30 μM ART, DHA, and ARTS in RPMI 1640 media (1% FBS). Photomicrographs of final wounds were taken at 24 and 48 h. Photomicrographs of initial wounds were taken by DMI 300B Leica at 100× magnification. Initial and final wound sizes were measured using Image Pro Plus 6.0 software.

### Transwell assay

Invasion analysis was performed by seeding A549 cells (1 × 10^5^) treated with 7.5, 15, or 30 μM ART, DHA, and ARTS into the upper chamber of a transwell apparatus coated with Matrigel and incubating the cells for 48 h. Cells were stained with crystal violet. The non-invasive cells were scraped off with cotton swabs, whereas the invasive cells stained with crystal violet were dissolved in DMSO for measurement.

### Immunofluorescence

Immunofluorescence was performed on A549 cells seeded on 15 mm confocal dish with 30 μM ART, DHA, ARTS treating for 48 h and fixed in cold 4% paraformaldehyde. The confocal dishes were incubated with primary antibodies. Detection of the primary antibodies was performed using 1:500 Alexa Fluor® conjugated secondary antibodies. The images were captured and analyzed using Leica TCS SP8 confocal microscope. All images were acquired using an identical acquisition time for all samples.

### Wnt5a siRNA transfection

A549 were plated into a 6-well plate with RPMI-1640 cell culture medium with 10% FBS. siRNA transfection was performed according to the manufacturer's protocol. Seven hours post-transfection, cells were treated with different concentrations (0, 7.5, 15, and 30 μM) of ART, DHA, and ARTS for 48 h. Cell lysates were analyzed for expression of Wnt5-a/b, β-catenin, Sox2, Oct3/4, N-cadherin, and E-cadherin proteins by Western blot analysis.

### Western blot

Total proteins were extracted from cells after treatments. Protein samples were separated on 8%–12% SDS-polyacrylamide gel electrophoresis. Blots were immunostained with primary and secondary antibodies. β-actin served as a loading control. Other detailed procedures were followed according to the literature [20].

### MMPs activity assay

MMPs activities were assessed using Amplite™ Universal Fluorimetric MMP Activity Assay Kit, according to standard protocols. In brief, 5,000 cells were seeded onto 24-well and then exposed to different concentrations (0, 7.5, 15, and 30 μM) of ART, DHA, and ARTS for 48 h. Finally, 50 μL of supernatants from each well was added to a 96-well plate for detection.

### Data analysis

Data were expressed as the mean ± standard deviation (SD) of at least three independent experiments. Statistical significance of the data was determined by one-way ANOVA using SPSS software. Statistical significance was considered at *p* < 0.05.

## SUPPLEMENTARY FIGURES


