# Supplementary material for: Artemisinin and its derivatives can significantly inhibit lung tumorigenesis and tumor metastasis through Wnt/β-catenin signaling
Source: Oncotarget. 2016 Apr 22;7(21):31413–28. doi: 10.18632/oncotarget.8920 (PMC5058767; doi:10.18632/oncotarget.8920)
Supplement: Supplementary file 1 [file oncotarget-07-31413-s001.pdf]

SUPPLEMENTARY FIGURES

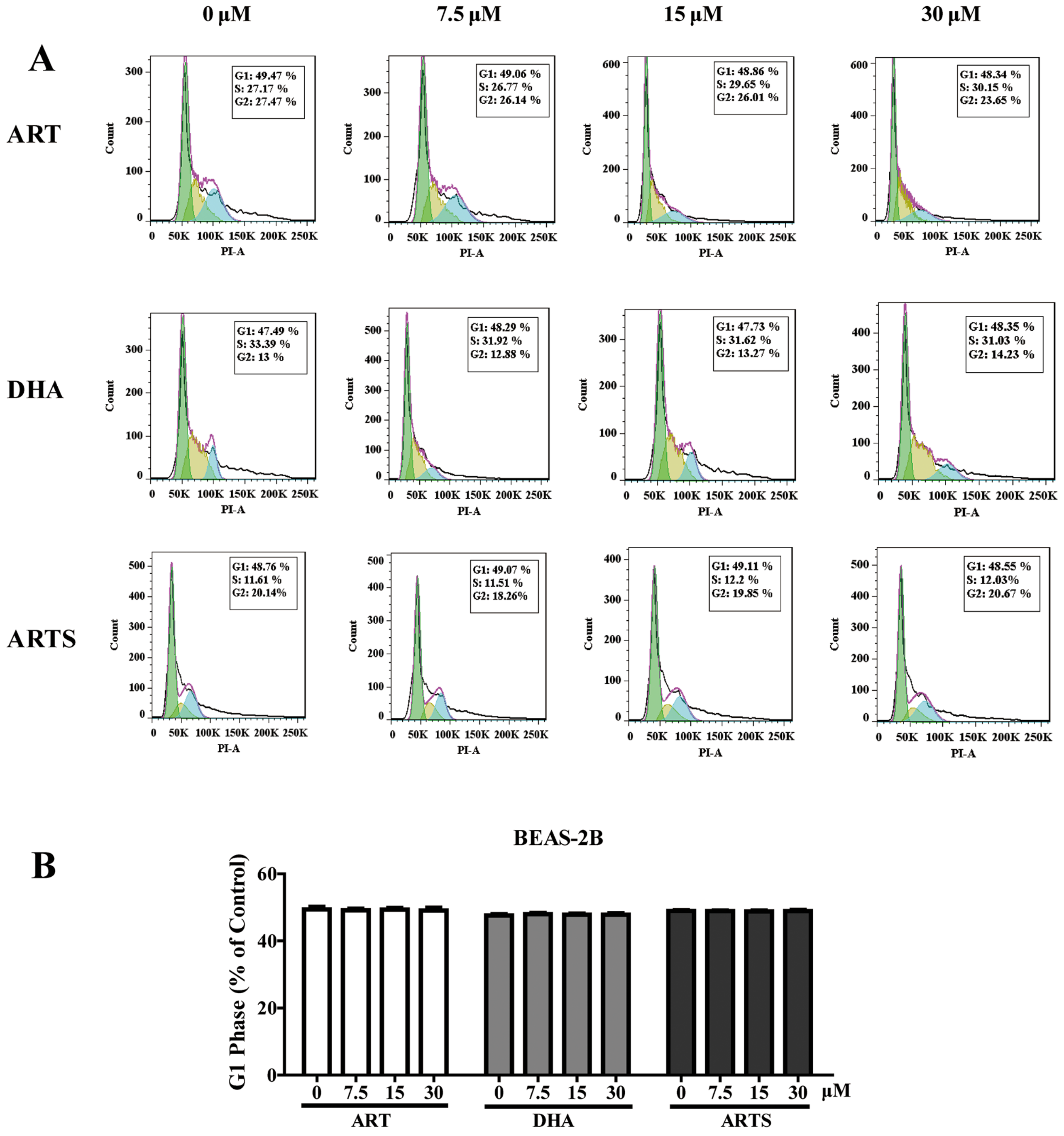

Supplementary Figure S1: Effects of ART, DHA, and ARTS on cell cycle progression of BEAS-2B. A. Cell cycle analysis was conducted for BEAS-2B with or without ART, DHA and ARTS. **B.** Percentages of BEAS-2B cells in the G1 phase after treatments with or without ART, DHA and ARTS.

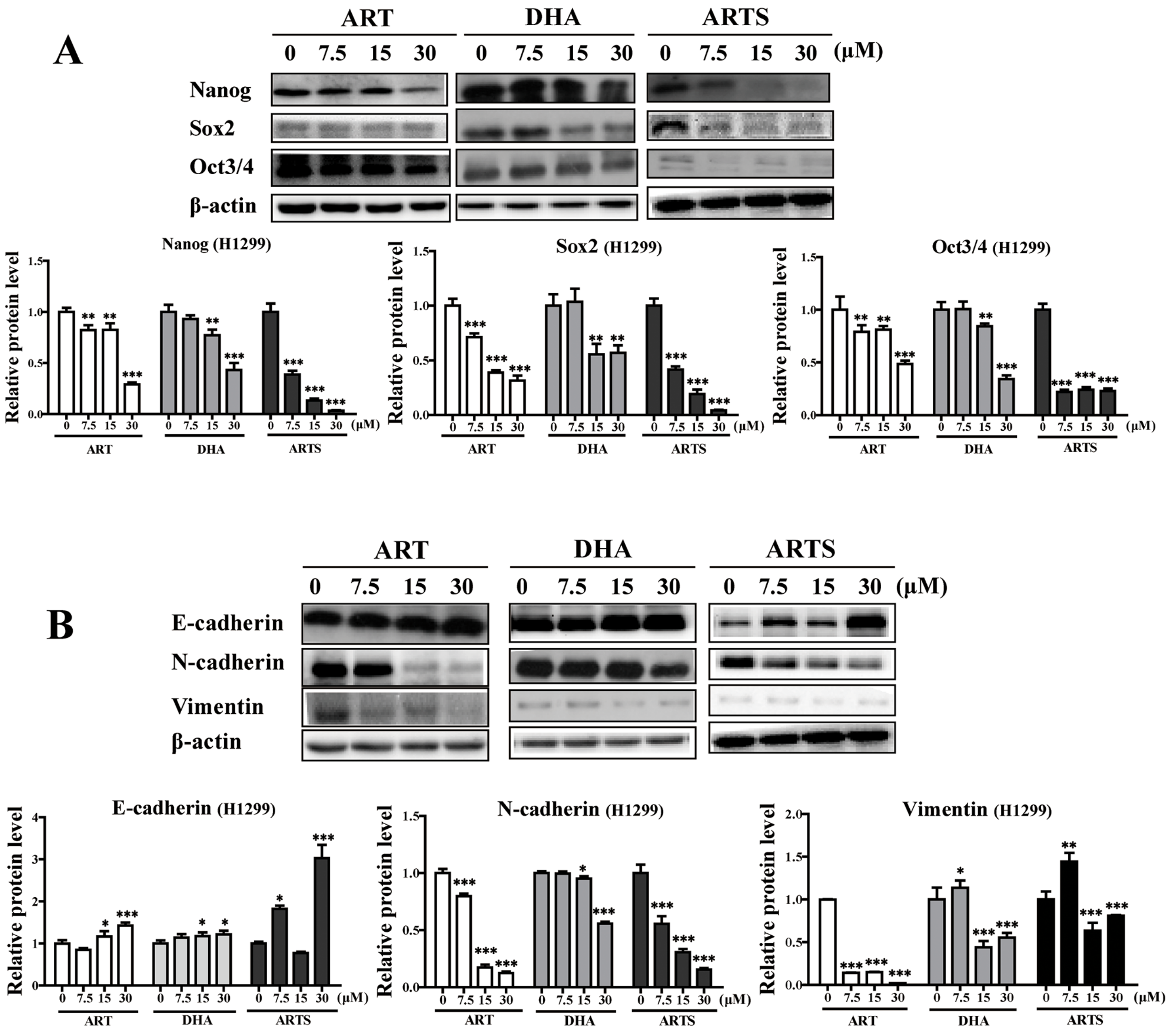

Supplementary Figure S2: ART, DHA, and ARTS through β-catenin-regulated EMT and CSCs of H1299 *in vitro*. Western blot analysis was performed for **A.** Nanog, Sox2, and Oct3/4 and **B.** E-cadherin, N-cadherin, and vimentin. Data showed were the means ± SD. **p* < 0.05, ***p* < 0.01, ****p* < 0.001, compared with the control group.
